# Stretchable Pressure Sensor with Leakage-Free Liquid-Metal Electrodes

**DOI:** 10.3390/s19061316

**Published:** 2019-03-15

**Authors:** Lunjia Zhang, Meng Gao, Ronghang Wang, Zhongshan Deng, Lin Gui

**Affiliations:** 1 A Key Laboratory of Cryogenics, Technical Institute of Physics and Chemistry, Chinese Academy of Sciences, Beijing 100190, China; zhanglunjia14@mails.ucas.ac.cn (L.Z.); mgao@mail.ipc.ac.cn (M.G.); wangronghang14@mails.ucas.ac.cn (R.W.); zsdeng@mail.ipc.ac.cn (Z.D.); 2 School of Future Technology, University of Chinese Academy of Sciences, 19 Yuquan Road, Shijingshan District, Beijing 100039, China

**Keywords:** liquid metal, stretchable pressure sensor, leakage-free electrode, double capacitor, parasitic capacitance

## Abstract

Nowadays, with the development of wearable devices, stretchable pressure sensors have been widely adopted in all kinds of areas. Most of the sensors aim to detect small pressure, such as fingertip tactile sensing, but only a few are focused on high-pressure sensing, such as foot pressure sensing during men’s walking. In this work, a liquid metal-based stretchable sensor for large-pressure measurement is investigated. This sensor is fully stretchable because it is made of soft materials. However, when the soft sensor is subjected to high pressure, the liquid metal easily leaks from microchannels because it maintains the liquid state at room temperature. We therefore propose to fabricate liquid metal-based leakage-free electrodes to handle the liquid-metal leak. Parametric studies are conducted to compare this sensor with liquid-metal-only electrodes and leakage-free electrodes. The leakage-free electrodes increase the measurement ranges from 0.18, 0.18, and 0.15 MPa to 0.44 MPa, with higher linearity and precision. The improvement in the liquid-metal electrode enables the sensors to work stably within 0.44 MPa pressure and 20% strain. In addition, we integrate two capacitors, namely, a working capacitor and a reference capacitor, into one sensor to reduce the influence of parasitic capacitance brought about by external interference. This stretchable capacitive sensor capable of working under a wide range of pressure with good repeatability, sensitivity, and linearity, exhibits great potential use for wearable electronics. Finally, the method for fabricating leakage-free electrodes shows great value for hyperelastic electronics manufacturing and micromachine technology.

## 1. Introduction

With development in soft wearable devices, stretchable sensors have been widely studied in recent years [1,2]. Elastic sensors are specially utilized in soft robots [3], healthcare monitoring [4], and even human implantable electronics [5]. Therefore, the materials used in soft sensors must have a good stretchability and excellent biocompatibility [6]. Among all materials for soft wearable electronics, PDMS (polydimethylsiloxane) and a eutectic alloy GaIn (Ga_75.5_In_24.5_ alloy, weight percentage, 75.5% Ga, 24.5% In, melting point 15.5 °C) [7,8] have gained increasing attention due to their superior properties in soft devices fabrication. The cured PDMS exhibits a good stretchability and biocompatibility as a substrate material [9],whereas the GaIn has a low toxicity and can maintain the liquid state at room temperature [10]. These features make the GaIn an ideal material for soft electrode fabrication, whereas the liquid-metal electrodes offer good robustness to fatigue and cracking compared with rigid-metal electrodes under stress or stretch [11,12]. In addition, GaIn soft electrodes can be fabricated more easily and economically than rigid electrodes, which are often synthesized by sputtering [13] or deposition [14,15].

In general, flexible pressure sensors have three major working types [16,17]: resistive [18], capacitive [19], and piezoelectric [20]. Among them, capacitive pressure sensors have been widely studied due to their advantages, such as their fast dynamic response, small energy consumption, and high repeatability [16,19,21]. However, capacitive sensors always have some problems, such as a nonlinear output and parasitic capacitance, which largely affect their performance [22].

Our work is focused on a GaIn liquid metal-based capacitive pressure sensor because of the biocompatibility, liquidity, and easy fabrication of the GaIn electrode. Many studies on liquid-metal capacitive pressure sensors have emphasized tactile sensing. These sensors have been developed with a high spatial resolution and sensitivity to detect a small force (usually less than 5 N or 0.1 MPa) [19,23,24,25]. However, stretchable sensors capable of measuring larger pressure (larger than 0.1 MPa) are also necessary; for example, the maximum pressure in foot-ground contact during men’s walking can exceed 0.1 MPa [26], and the pressure in tire-ground contact during car rolling can be larger. Hence, stretchable sensors with a large measurement range show great potential in a wide range of applications. However, reports on the flexible sensors for large pressure sensing are relatively rare [27,28,29,30]. Yeo et al. [28] developed a liquid metal-based resistive sensor that could sense various levels of pressure, such as foot stomping, chair rolling, and car rolling; however, this sensor did not have a linear output within the whole sensing range, and the fabrication of the screen-printed silver electrode of the sensor was costly and complicated as screen printing technology requires multiple procedures [31]. In addition, the silver electrode may be vulnerable to stretching as thin metal films can be easily detached from soft substrates upon mechanical stress [32]. Ali et al. [29] reported a liquid metal-based capacitive sensor that could work under large pressure; nonetheless, they neglected the capacitance change under pressure ranging from 0 MPa to 0.25 MPa. Narakathu et al. [30] fabricated flexible capacitive pressure sensors on the basis of silver nanoparticle ink by using conventional printing technology. The maximum detectable pressure of the sensor was 18 MPa, but its smallest detectable pressure was ~800 kPa. Although all the sensors mentioned above could sense large pressure, they still had limitations, such as a complicated fabrication or low resolution.

For a liquid electrode-based stretchable sensor, a wide working range necessitates good packaging because liquid electrodes will leak once the applied pressure exceeds a certain value. This leak will change the effective facing area of capacitive sensors or the original resistance of resistive sensors, thereby limiting the working range and lowering the repeatablity. Lim et al. [17] enhanced the bonding strength of the flexible sensor to prevent the leakage of the graphene oxide (GO) nanosuspension liquid-based electrode by oxygen plasma and UV ozone treatment; however the sensor required a complicated and costly deposition procedure to fabricate leading electrodes, which were used to connect the sensor with external devices. A high bonding strength is always required when the electrodes are directly deposited or sputtered on soft substrates, thereby increasing the fabrication complexity. In addition, some studies reported the liquid-metal necking and breaking within a single channel under stretch [33] and jets of the liquid-metal break-up induced by interfacial tension [34]; however, very few have focused on the liquid-metal leakage and its solutions.

In this work, we present a simple way to fabricate a GaIn-based double-capacitor pressure sensor. To prevent GaIn leak under large pressure, we develop a new method to make leakage-free electrodes by introducing an alloy of Bi_32.5_In_51_Sn_16.5_ (weight percentage, 32.5% Bi, 51% In, 16.5% Sn, melting point 60 °C) into the electrodes. We conduct experiments to prove that the leakage-free electrodes can effectively increase the measurement range, mechanical stability, and linearity of the sensor compared with those of GaIn-only electrodes. In addition, we confirm the ability of the double-capacitor structure to reduce parasitic capacitance brought about by external interference. Overall, this work presents a simple method to fabricate a stretchable pressure sensor with leakage-free electrodes that measures large pressure, reduces parasitic capacitance, and has a relatively high sensitivity.

## 2. Materials and Methods

### 2.1. Design and Fabrication

**Figure 1:**
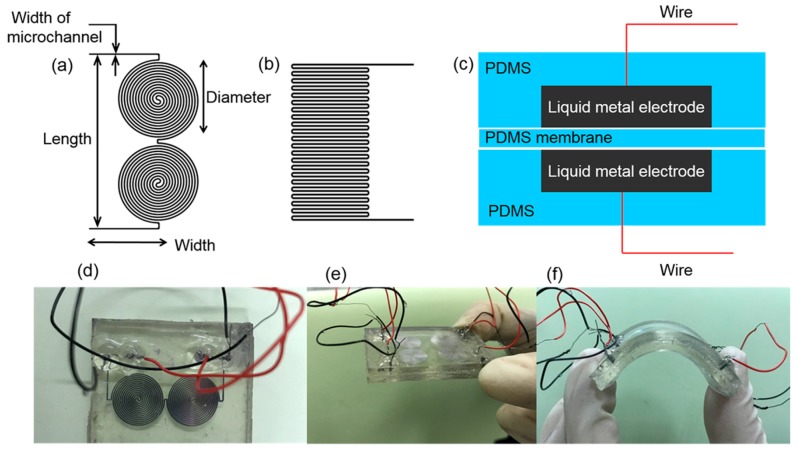
Diagrams of the stretchable capacitive pressure sensor. (**a**,**b**) Shape of the microchannels; (**c**) schematic diagram of the sensor; (**d**) vertical view of the sensor; (**e**) original state of the sensor; (**f**) the stretchable sensor under bend.

The microchannels as shown in Figure 1a,b were patterned on PDMS (SYLGARD 184 Silicone Elastomer Kit, Dow Corning, Midland, MI, USA) slabs by using soft-lithography technology. The liquid PDMS was made by manually mixing the prepolymer and the curing agent at a weight ratio of 10:1 using a glass rod. This mixture was then vacuum evacuated for 1 h in a vacuum drying oven (DZF, Yiheng, Shanghai, China). Finally, the liquid PDMS was cured on a hot plate (EH20B, Labtech, Beijing, China) at 65 °C for 2.5 h. With the microchannels winded, the sensor had a working area of only several square centimeters [33]. After fabricating the PDMS slabs, we bonded two patterned PDMS slabs face-to-face with a PDMS thin membrane at the middle. The PDMS membrane was fabricated as follows: (a) uncured PDMS was first spin coated on a silicon wafer, (b) and then baked at 75 °C for 30 min. Afterwards, we injected GaIn liquid metal into the microchannels to form liquid-metal electrodes. The GaIn liquid metal was fabricated as follows: gallium and indium were melted at 150 °C at a weight ratio of 75.5:24.5 in a dry box (Yiheng, Shanghai, China) for 2 h. We then stirred them using a glass rod for 5 min until all the metal was blended. Afterwards, we placed them back into the dry box for 30 min. Finally, we cooled the liquid metal down to room temperature. As shown in Figure 1c, a pair of liquid-metal electrodes was symmetrically formed and bonded to a PDMS membrane face-to-face. The PDMS exhibits a good stretchability after cured, and the GaIn can always maintain the liquid state at room temperature. As a result, the whole sensor is fully stretchable. This stretchable sandwich-structured sensor functions similar to a parallel-plate capacitor. A pair of silver plated copper wires (WIRE WRAPPING WIRE, B-30-1000, Onlyou, Shenzhen, China) was inserted into the inlet/outlet holes to maintain contact with the liquid-metal electrodes, thereby enabling the sensor to be connected to external devices. Figure 1d shows an as-made soft sensor that adopted Figure 1a as the shape of its electrodes. A small force can lead the sensor to deform due to the flexibility of the sensor [35]. As shown in Figure 1e,f, after the force was released, the sensor could return to its original state without any change.

### 2.2. Working Principles

Because the stretchable pressure sensor in our work can be regarded as a parallel-plate capacitor, the capacitance can be given as [21,36]

(1) $$C=\frac{\varepsilon_{0\cdot }\varepsilon_{r\cdot S}}{d},\;$$

where $\varepsilon_{0}=8{.85\times 10}^{12}\;F/m$ is the vacuum dielectric constant, $\varepsilon_{r}$ is the relative dielectric constant of the dielectric medium in the capacitor, S is the facing area of the electrode, and d is the vertical distance between the two electrodes. When the sensor is subjected to pressure, it will be stretched in different directions. The deformation causes the facing area of the electrodes to increase and the distance between them to decrease; as a result, the capacitance will increase as expressed in Equation (1). A capacitance-pressure curve can be drawn by measuring a sequence of capacitance values corresponding to different pressures, which will be discussed in the next section.

**Figure 2:**
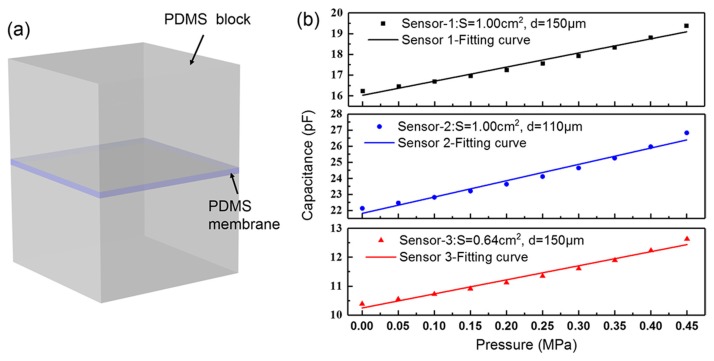
Simulation of the PDMS (polydimethylsiloxane)-based sensors under pressure. (**a**) Simplified geometric model of the capacitive sensor; (**b**) capacitance change of the sensors with different dimensions under pressure.

As shown in Figure 2a, we used COMSOL 5.3 to make an approximate simulation based on actual dimensions of the soft sensors. The height of the PDMS block is 2.5 mm. The shape of the effective facing area of the top and the bottom electrode is simplified to a square according to the actual area of the liquid metal-filled microchannel. We simulated the capacitance change of the PDMS- based sensors under increasing pressure. Different facing areas *S* and vertical distances *d* are investigated in Figure 2b. The capacitance change shows a linear correlation, with the pressure ranging from 0 to 0.45 MPa (*R*^2^ = 0.98, *R*^2^ is the regression coefficient). A discrepancy exists between the simulation and the measured values because parasitic capacitance is unavoidable and hard to estimate. Besides, the misalignment between the top and the bottom electrode (~50 μm) also adds to the discrepancy.

### 2.3. Fabrication of GaIn-BiInSn Leakage-Free Electrodes

**Figure 3:**
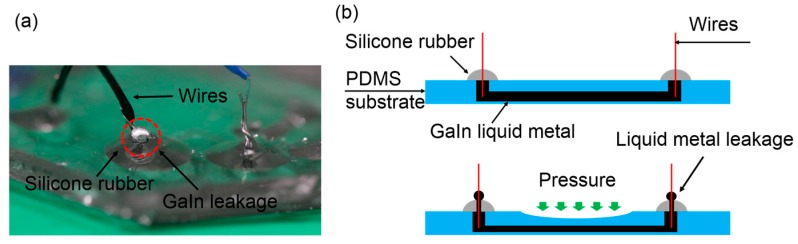
Leakage of the GaIn liquid metal. (**a**) Enlarged view of the liquid-metal leakage; (**b**) liquid metal leaks from the microchannel under large pressure.

**Figure 4:**
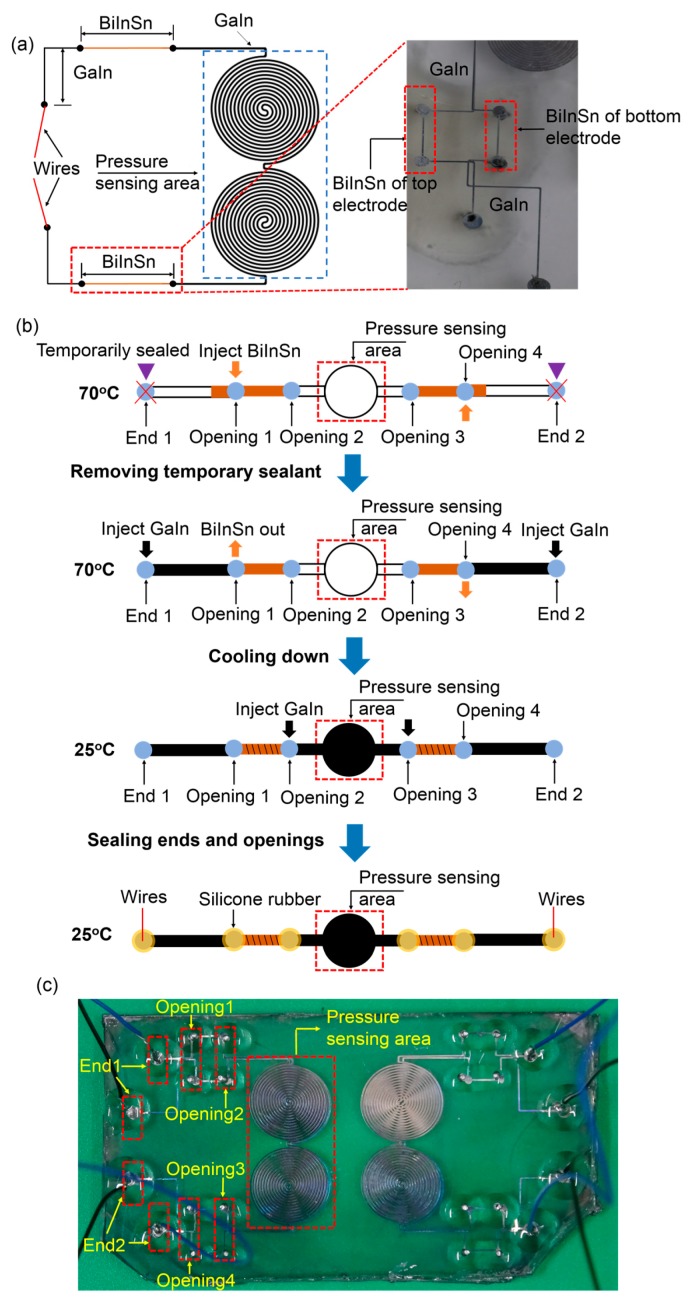
(**a**) Structure of the GaIn-BiInSn leakage-free electrode; (**b**) fabrication process of the leakage-free electrode; (**c**) fabricated stretchable capacitive sensor with the leakage-free electrode.

The liquid-metal electrodes merely composed of the GaIn alloy easily failed due to leak of the GaIn, as shown in Figure 3a, when applying pressure that exceeded the measurement range of the sensor. Although we sealed the inlets and outlets using RTV silicone rubber (705, Nanda, Nanjing, China), the liquid metal leaked along the leading wires, as shown in Figure 3b. Therefore, we proposed a new GaIn-based leakage-free electrode to widen the measurement range of the sensor by introducing an alloy of Bi_32.5_In_51_Sn_16.5_. The BiInSn was fabricated as follows: bismuth, indium, and tin were melted at 260 °C at a weight ratio of 32.5:51:16.5 in a dry box (Yiheng, Shanghai, China) for 2.5 h. We then stirred them using a glass rod for 20 min until all the metal was blended. Afterwards, we placed them back into the dry box for 1 h. Finally, we cooled the alloy down to room temperature. We filled a small segment of the microchannels (0.5 cm in length) with the BiInSn near the ends (inlet and outlet) of the electrode microchannels, as shown in Figure 4a. The BiInSn maintained the solid state at room temperature. Hence, the segments filled with the BiInSn worked as a plug of the electrode to block the GaIn alloy of the pressure sensing area from leaking when the sensor was subjeted to large pressure.

The fabrication of the leakage-free electrode is illustrated in Figure 4b: we initially temporarily sealed end 1&2 and injected the BiInSn (liquid state) from opening 1(4) to opening 2(3) at 70 °C on a hot plate (EH20B, Labtech, Beijing, China). However, a small amount of the BiInSn might flow towards end 1(2) during the process. We then removed the temporary sealants and injected the GaIn from end 1(2) to opening 1(4) to fill the microchannels. Meanwhile, the GaIn pushed the small amount of BiInSn to flow out from opening 1(4). Afterwards, we cooled the BiInSn electrode to room temperature (25 °C). However, the BiInSn shrank inside the microchannels when it turned into the solid phase, which usually causes some defects inside the BiInSn electrode [37]. These defects made the electrode easy to break. We therefore injected the GaIn from opening 2 or 3. During the process, the majority of the GaIn filled the pressure sensing area, and naturally, a tiny amount of GaIn flowed into the BiInSn-filled microchannels to fill these defects. The GaIn filled the pressure sensing area completely due to the blocking of the BiInSn. Finally, the wires were inserted into end 1&2, and we used the silicone rubber to seal all the ends and openings. The fabricated sensor is shown in Figure 4c. The leakage-free electrode is compact and is easy to integrate into stretchable devices. Compared with inserting wires into the holes to prevent the GaIn leak, this method is especially practical for very thin stretchable sensors because the wires easily fall down and the GaIn will still leak along the wires.

**Figure 5:**
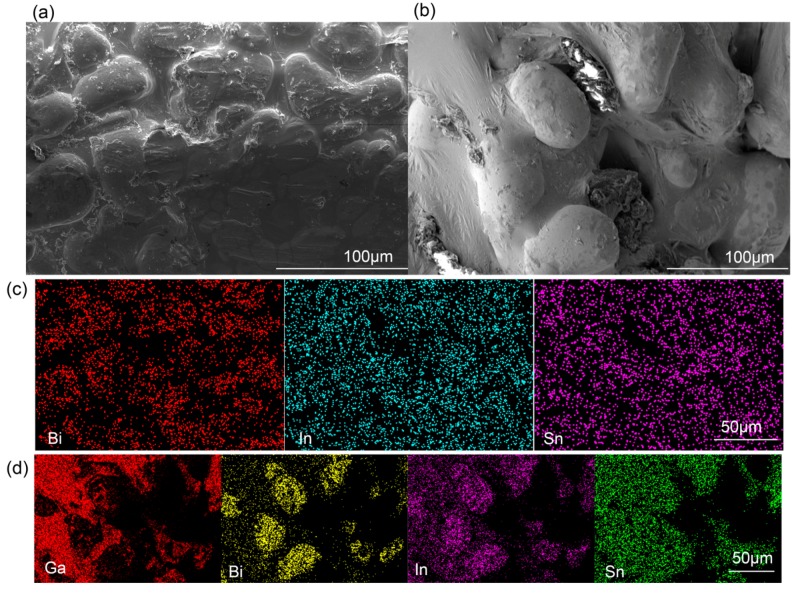
Electron microscopic structure of the BiInSn electrode (**a**) before and (**b**) after the introduction of the GaIn; elemental distribution of the BiInSn electrode (**c**) before and (**d**) after the introduction of the GaIn.

Figure 5 shows the interface property and composition of the leakage-free electrode. The microscopic structure of the BiInSn electrode before and after the introduction of the GaIn were observed by ESEM (environmental scanning electron microscope, QUANTA FEG 250, Hillsboro, OR, USA), as presented in Figure 5a,b, which are the cross sections of the solid region (the BiInSn) within the microchannel. The surface of the BiInSn electrode is very rough, as the sags and crests indicate. The elemental distributions of the BiInSn electrode before and after the introduction of the GaIn are shown in Figure 5c,d obtained by EDS (Energy Dispersive Spectrometer, Oxford Instruments, Oxford, UK). The introduced GaIn filled cracks in the BiInSn electrode and wrapped its local surface, as represented by the wrinkles in Figure 5b. Therefore, the leakage-free electrode takes advantage of the fluidity of the GaIn and the rigidity of the BiInSn at room temperature.

### 2.4. Measurement Setup and Method

**Figure 6:**
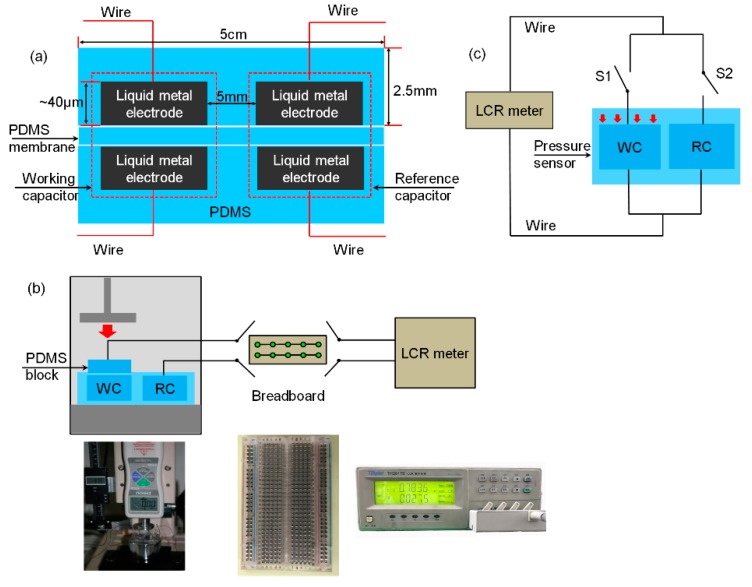
(**a**) Structure of the double-capacitor sensor; (**b**) measurement setup of the experiment; (**c**) equivalent circuit of the measurement.

The stretchable sensor consists of two identical capacitors, i.e., a working capacitor (WC) and a reference capacitor (RC), to reduce parasitic capacitance brought about by external interference. Its dimensions are marked as shown in Figure 6a. The WC was used to detect pressure, whereas the reference capacitor was used to detect the parasitic capacitance. Figure 6b shows the experimental setup for the double-capacitor pressure sensor. A PDMS block (*L* × *W* × *H*: 2.6 cm × 1.3 cm × 2.5 mm) was placed on the WC. In this manner, the pressure was only exerted on the WC by a punching machine (ZQ-21B-1, Zhiqu, Dongguan, China). We used a breadboard to control the circuit manually and a LCR meter (TH2817B, Tonghui, Changzhou, China) to measure capacitance values. The WC and the RC were controlled by two switches in the circuit equivalently, as shown in Figure 6c.

**Figure 7:**
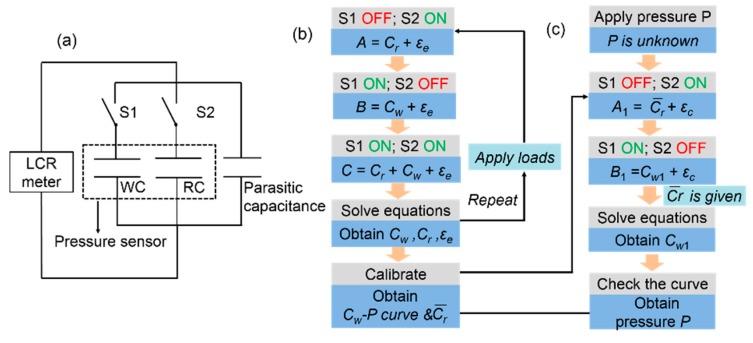
Control scheme to reduce parasitic capacitance. (**a**) Equivalent circuit of the control scheme; (**b**) flowcharts of the calibration process; (**c**) flowcharts of the measuring process.

Figure 7 plots the control scheme of the double-capacitor pressure sensor to reduce the parasitic capacitance. The method includes a calibration process and a measuring process. Figure 7a,b illustrate the calibration process. By solving the equations in Figure 7b, we are able to obtain $C_{r}$, $C_{w}$ and $\varepsilon_{e}$ which are expressed in Equation (2):

(2) $$\begin{array}{c} C_{w}=C-A,\\ C_{r}=C-B,\\ \varepsilon_{e}=A-C_{r}, \end{array}$$

where *A*, *B*, and *C* are the measured values of the reference capacitance, the working capacitance, and the capacitance when the WC and the RC are parallel-connected, respectively, during the calibration process; $C_{w}$ and $C_{r}$ are the true values of the working capacitance and the reference capacitance, respectively; and $\varepsilon_{e}$ is the parasitic capacitance brought about by external interference. The calibration curve between $C_{w}$ and pressure is obtained after the calibration process. Besides, a group of reference capacitances $C_{r}$ is also obtained during the calibration process, and the average of them is regarded as the true value of the reference capacitance. We note it as $\bar{C_{r}}$.

Given the calibration curve between the pressure and the WC, the measuring process shown in Figure 7a,c is targeted at obtaining the true value of the WC when different pressure is applied though noise (parasitic capacitance) is mixed. The measuring process is similar to the calibration process, except $C_{r}\;$ is known as $\bar{C_{r}}$ after the calibration process. As shown in Figure 7a,c, the current working capacitance is obtained according to Equation (3):

(3) $$\begin{array}{c} C_{w1}=B_{1}-A_{1}+\bar{C_{r}},\\ \varepsilon_{c}=A_{1}-\bar{C_{r}}, \end{array}$$

where $C_{w1}$ is the true value of the working capacitance under pressure *P*; $B_{1}$ and $A_{1}$ are the corresponding measured values when the WC and the RC are connected in the circuit alone, respectively; and $\varepsilon_{c}$ is the parasitic capacitance under the current measuring environment. On this basis, the pressure applied to WC can be found out in accordance with the calibration curve as we have already obtained $C_{w1}$.

## 3. Results and Discussion

We fabricated three different sensors in the experiments. Their parameters are listed in Table 1. Figure 1a shows the pattern of the electrodes of the sensors. Results and analysis are discussed as follows.

### 3.1. Performance Analysis

We compared the sensors with the GaIn-only electrode (A_1_, B_1_, C_1_) and those with the leakage-free electrode (A, B, C) when they were subjected to a wide range of pressure. The sensors were attached to the flat support plate of the punching machine, as shown in Figure 6b. Loads were gradually applied on the sensor with increments of 0.5 kg. Each sensor was pressured three times, and its capacitance was measured under the frequency of 50 kHz.

**Figure 8:**
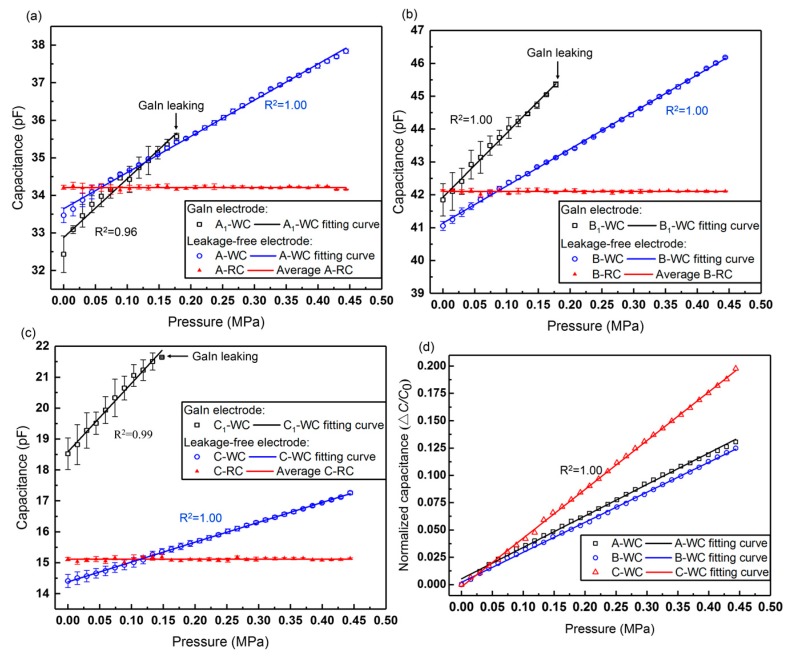
(**a**–**c**) Pressure response curves of the GaIn and the leakage-free electrode-based sensors. (**d**) Sensitivities of Sensor A, B, and C with different electrodes.

As seen in Figure 8, the working capacitances of both types of sensors increase linearly with the pressure. The results show accordance with the simulation results in Section 2.2. However, the GaIn electrode-based sensors A_1_, B_1_, and C_1_ have displayed liquid-metal leak at the pressure of 0.18, 0.18, and 0.15 MPa, respectively. By contrast, all the leakage-free electrode-based sensors A, B, C can exhibit a linear output within the pressure of 0.44 MPa, without the liquid-metal leak. Their measurement ranges and linear coefficients are listed in Table 2. In addition to the larger measurement range and the higher linearity, the leakage-free electrode can also improve the precision of the sensors, as indicated by the error bars in Figure 8.

Figure 8d plots the normalized capacitance change of the working capacitances with the pressure. The sensitivity of the sensor is defined as Equation (4):

(4) $$S=\frac{\Delta C/C_{0}}{P}=\frac{\left(C_{loaded}-C_{unloaded}\right)/C_{unloaded}}{P},$$

where $C_{unloaded}$ is the initial capacitance of the sensor under no pressure, and $C_{loaded}$ is the corresponding capacitance when different loads are applied on the WC. Therefore, the slope of the fitting curve is calculated as the sensitivity of the sensor. Thus, the sensitivities of Sensor A, B, and C are 0.29, 0.27, and 0.45 MPa^−1^, respectively. Table 3 compares different PDMS dielectric-based flexible pressure sensors. The sensor in our work shows a relatively high sensitivity under a large pressure.

### 3.2. Leak Test

#### 3.2.1. Pressure Limit Test

**Figure 9:**
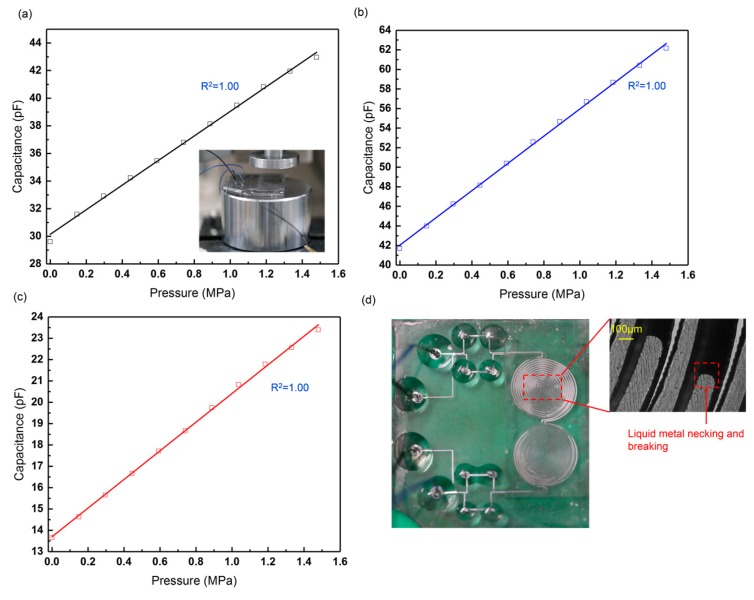
Capacitance change of leakage-free electrode-based (**a**) Sensor A, (**b**) Sensor B, and (**c**) Sensor C under increasing pressure from 0 to 1.48 MPa; (**d**) electrode failure of the stretchable sensors.

Figure 9a–c plot the capacitance change of the three sensors under extremely large pressure (1.48 MPa (50 kg), the maximum pressure that the punching machine can give). The capacitances of the sensors increase linearly with the pressure, and no liquid-metal leakage was observed during the press test. However, the electrodes were broken, as seen in Figure 9d. Large mechanical deformation will cause necking and breaking of the liquid-metal electrode [33], thereby lowering the life of the stretchable sensors and causing electrode failure. The necking and breaking can also be explained by the GaIn leak as some liquid metal might flow into the GaIn-BiInSn structure irreversibly under extremely high pressure, although the leakage cannot be observed at the inlet or the outlet. In accordance with our experiments, the three stretchable sensors displayed an optimum performance within 0.44 MPa, as shown in Section 3.1, because the linearity and the repeatability of them declined when the pressure exceeded 0.44 MPa.

**Figure 10:**
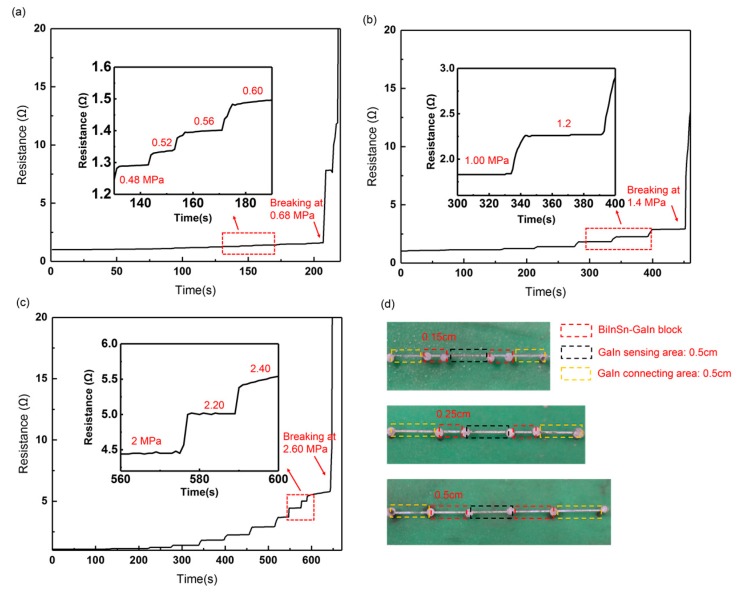
Resistance change of a typical leakage-free electrode with a (**a**) 0.15 cm, (**b**) 0.25 cm, (**c**) 0.5 cm long GaIn-BiInSn structure; (**d**) diagram of typical leakage-free electrode.

Since the GaIn-BiInSn structure can effectively reduce the liquid-metal leak, which seriously limits the measurement range of the sensor, we quantitatively studied how the length of the GaIn-BiInSn structure could affect the measurement range. Figure 10 shows the resistance change of the typical leakage-free electrodes under increasing pressure. Three typical leakage-free electrodes with different GaIn-BiInSn lengths were fabricated as shown in Figure 10d, and the typical electrode consists of a GaIn sensing area, two GaIn-BiInSn blocks, and two GaIn connecting areas. The lengths of the GaIn sensing area and the GaIn connecting area are both 0.5 cm. The results in Figure 10a–c show that the electrodes with a 0.15 cm, 0.25 cm, and 0.5 cm long GaIn-BiISn structure break at 0.60 MPa, 1.40 MPa, and 2.60 MPa, respectively. This is because a longer GaIn-BiInSn structure has a higher resistance to the GaIn leak, which increases the measurement range of the electrode.

#### 3.2.2. Stretch Test

**Figure 11:**
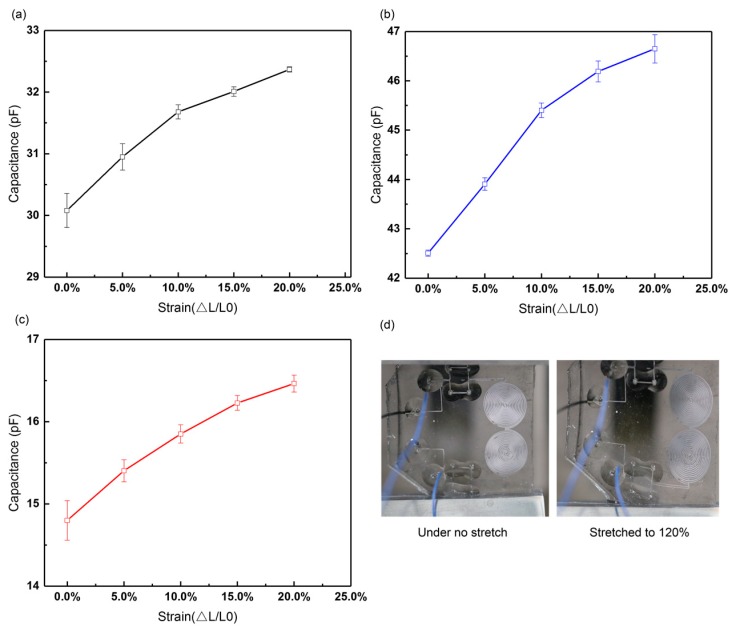
Strain response curves of leakage-free electrode-based (**a**) Sensor A, (**b**) Sensor B, and (**c**) Sensor C; (**d**) stretchable sensors under stretch.

We conducted stretch tests to display the stretchability of the sensors. Each test was repeated three times. Figure 11 shows that the sensors can be stretched to 120% their original length without electrode breaking. As seen in the figure, the sensitivities of Sensor A, B, and C are 0.53, 0.68, and 0.54, respectively, when the strain ranges within 10%, and then decrease to 0.22, 0.27, and 0.38, respectively, when the sensors are stretched from 110% to 120%.

#### 3.2.3. Ohmic Test

**Figure 12:**
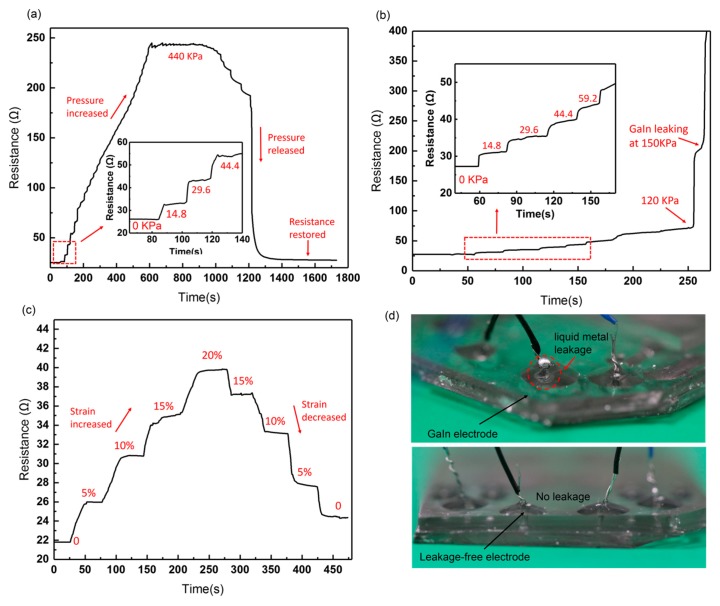
Real-time resistance response curves of (**a**) the leakage-free electrode under pressure from 0 to 0.44 MPa; (**b**) the GaIn-only electrode under pressure from 0 to 0.15 MPa; (**c**) the leakage-free electrode under strain from 0 to 20%; (**d**) liquid metal of the GaIn-only electrode leaks at 0.15 MPa.

**Figure 13:**
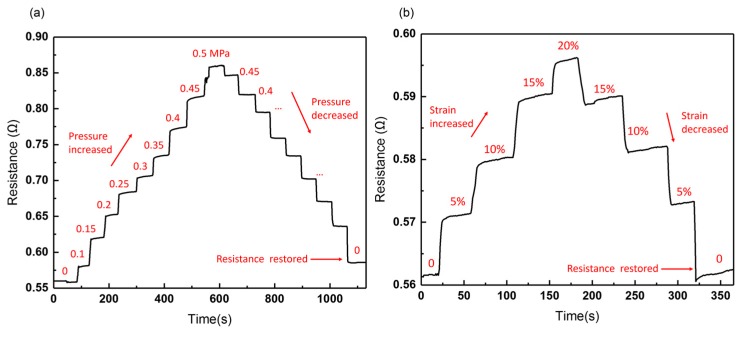
Real-time resistance change of the GaIn-BiInSn filled region under (**a**) pressure from 0 to 0.5 MPa and (**b**) strain from 0 to 20%.

In order to explore the stability of the leakage-free electrode, we initially measured the resistance change of the whole electrode in response to increasing pressure and stretch. As seen in Figure 12a, the maximum pressure applied to the leakage-free electrode is up to 0.44 MPa. After the pressure is released, the resistance of the electrode can basically be restored to its initial value. By contrast, Figure 12b shows that the resistance of the GaIn-only electrode increases abruptly at 0.12 MPa, and the GaIn leakage can be observed at 0.15 MPa, as shown in Figure 12d. Figure 12c illustrates that the resistance of the leakage-free electrode can be restored, even if it is stretched to 120% its original length (original length: ~30 mm). We then specially measured the resistance change of the GaIn-BiInSn filled region (0.5 cm long and 200 μm wide), as shown in Figure 13. The results display that the resistance of the compounded region can be restored after it is pressured under 0.5 MPa and stretched to 120% its original length, which possibly indicates that there is no interfacial failure between GaIn and BiInSn in the compounded region when the pressure and the strain range are within 0.50 MPa and 20%, respectively. The ohmic test fully exhibits the good mechanical stretchability and stability of the leakage-free electrode.

### 3.3. Anti-Interference Test

According to the steps in Figure 7, we conducted an experiment to explore the ability of the double-capacitor sensor to reduce parasitic capacitance brought about by external interference. We used a metal plate to cover the measuring probe for mimicking real-life interference, which was treated as a part of parasitic capacitance in this experiment.

**Figure 14:**
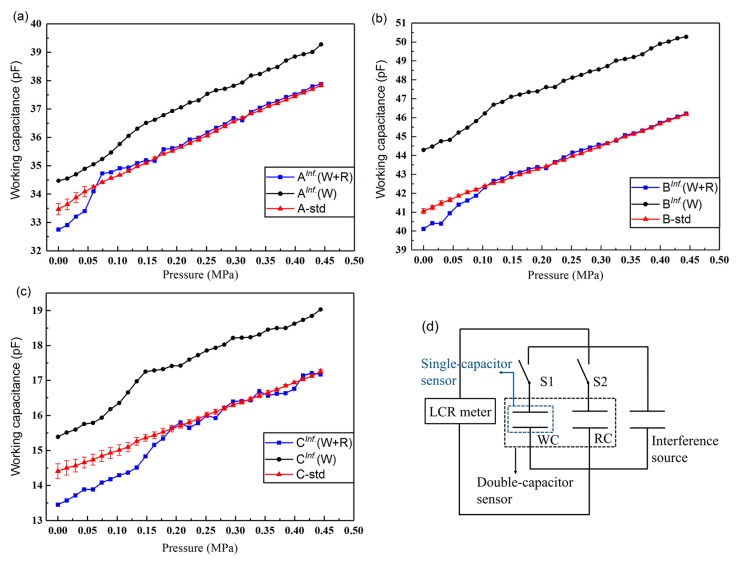
Comparison of reducing parasitic capacitance between the double-capacitor sensor and the single-capacitor sensor. The superscript “*Inf*.” means the sensor was under the external interference; “Std.” means the benchmark of the sensor measured under a standard testing environment without the interference; (W+R) means the double-capacitor sensor; (W) means the single-capacitor sensor. (**a**,**b**,**c**) The outputs of Sensor A, B, and C in the anti-interference test; (**d**) equivalent circuit of the anti-interference test.

As seen in Figure 8a–c, the reference capacitances of the leakage-free electrode-based sensors remain almost unchanged under different loads, as indicated by the red curves, which satisfies our control scheme in Section 2.4, where the RC was free of the applied pressure. Therein, the true values of the reference capacitances of Sensor A, B, and C are 34.21, 42.10, and 15.11 pF, respectively. Figure 14a–c compare the performance of the double-capacitor sensor (the WC and the RC, W+R) and the single-capacitor sensor (the WC only, W) in the anti-interference test. The equivalent circuit is shown in Figure 14d. Equation (5) gives the expression that can be used to calculate the capacitance deviations of the sensors from their standard values when the double-capacitor sensor and the single working-capacitor sensor are under the same interference. Herein, a lower *D* value means a higher ability to reduce parasitic capacitance.

(5) $$\begin{array}{c} D_{W+R}=\sum_{i=1}^{n}\left[\frac{C_{i,W+R}^{Inf.}-C_{i}^{Std.}}{C_{i}^{Std.}}\right]/n,\\ D_{W}=\sum_{i=1}^{n}\left[\frac{\left|C_{i,W}^{Inf.}-C_{i}^{Std.}\right|}{C_{i}^{Std.}}\right]/n, \end{array}$$

where *n* is the *n*-th applied load, the superscript “*Inf*.” Means that the sensor was under the external interference, and “*Std*.” means the benchmark of the sensor; for example, $C_{1,W+R}^{Inf.}$ is the working capacitance of the double-capacitor sensor under the interference when the first load (*i* = 1) is exerted. Therefore, the deviation values of Sensor A, B, and C with the double capacitors under the interference are 0.54%, 0.52%, and 2.31%, respectively. By contrast, the deviation values of those with the single working capacitor under the interference are 3.44%, 9.06%, and 10.08%, respectively.

The results reveal that the performance of double-capacitor sensors is better than that of single-capacitor sensors in the anti-interference experiment. The ability to reduce the parasitic capacitance is positively related to the magnitude of the capacitance.

## 4. Conclusions

In this work, we have fabricated a liquid metal-based double-capacitor pressure sensor with GaIn-BiInSn leakage-free electrodes. The sandwich-structured sensor consists of PDMS substrates, a pair of liquid metal-based electrodes, a PDMS membrane, and silver plated copper wires. In order to solve the problem of GaIn liquid-metal leaking, a leakage-free electrode has been fabricated in our work by introducing BiInSn. The results show that the GaIn-BiInSn electrodes can increase the measurement ranges from 0.175, 0.175, and 0.340 MPa to 0.440 MPa, respectively, without GaIn leaking. Meanwhile, these electrodes improve the linearity and the precision of the sensor compared with the GaIn-only electrodes. The sensitivities of the leakage-free electrode-based sensors are 0.29, 0.27, and 0.45 MPa^−1^, respectively. The leak test shows that the leakage-free electrode can display a very stable performance when the pressure and the strain ranges are within 0.44 MPa and 20%, respectively. In the anti-interference test, we prove that the double-capacitor sensor can effectively improve the ability to reduce parasitic capacitance compared with the single-capacitor sensor. Additionally, the ability to reduce parasitic capacitance is positively related to the magnitude of the capacitance. The stretchable capacitive pressure sensor capable of working under a wide range of pressure with a good repeatability, sensitivity, and linearity, exhibits great potential use for wearable electronics. Furthermore, the fabrication method of the GaIn-BiInSn leakage-free electrodes is a promising way to make soft and stable microelectrodes and facilitates the practical application of the fluidity of GaIn and the rigidity of BiInSn.

## Figures and Tables

**Table 1:** Geometric dimensions of capacitors.

Sensor	Width of Microchannels (μm)	Thickness of Dielectric (μm)	Gap between Microchannels (μm)	Length (cm)	Width (cm)	Diameter (cm)
A	200	150	200	2.5	1	1.2
B	200	110	200	2.5	1	1.2
C	150	150	100	1.8	1	0.8

**Table 2:** Comparison of the GaIn and the leakage-free electrode-based sensors.

Sensor	GaIn			Leakage-Free Electrode		
	A_1_	B_1_	C_1_	A	B	C
Measurement range (MPa)	0.18	0.18	0.15	0.44	0.44	0.44
Linear coefficient (*R*^2^)	0.96	1.00	0.99	1.00	1.00	1.00

**Table 3:** Comparison of PDMS dielectric-based capacitive pressure sensors.

	Types	Sensitivity	Measurement Range
Ali et al. [29]	GaIn liquid metal	0.11% MPa^−1^	0.25–1.1 MPa
Narakathu et al. [30]	Silver nanoparticle	0.02 MPa^−1^	0.8–18 MPa
Hong et al. [38]	Silver nanowire	0.35 KPa^−1^	2.5–4.5 KPa
This work	GaIn-BiInSn	0.45 MPa^−1^	0–0.44 MPa
